# Large-scale metagrating complex-based light field 3D display with space-variant resolution for non-uniform distribution of information and energy

**DOI:** 10.1515/nanoph-2022-0637

**Published:** 2023-01-13

**Authors:** Jianyu Hua, Fengbin Zhou, Zhongwen Xia, Wen Qiao, Linsen Chen

**Affiliations:** School of Optoelectronic Science and Engineering & Collaborative Innovation Center of Suzhou Nano Science and Technology, Soochow University, Suzhou 215006, China; Key Lab of Advanced Optical Manufacturing Technologies of Jiangsu Province & Key Lab of Modern Optical Technologies of Education Ministry of China, Soochow University, Suzhou 215006, China; SVG Optronics, Co., Ltd, Suzhou 215026, China

**Keywords:** light field 3D display, metagrating complex, non-uniform distribution, space-variant resolution

## Abstract

Glasses-free three-dimensional (3D) display has attracted wide interest for providing stereoscopic virtual contents with depth cues. However, how to achieve high spatial and angular resolution while keeping ultrawide field of view (FOV) remains a significant challenge in 3D display. Here, we propose a light field 3D display with space-variant resolution for non-uniform distribution of information and energy. The spatial resolution of each view is modulated according to watching habit. A large-scale combination of pixelated 1D and 2D metagratings is used to manipulate dot and horizontal line views. With the joint modulation of pixel density and view arrangement, the information density and illuminance of high-demand views are at most 5.6 times and 16 times that of low-demand views, respectively. Furthermore, a full-color and video rate light field 3D display with non-uniform information distribution is demonstrated. The prototype provides 3D images with a high spatial resolution of 119.6 pixels per inch and a high angular resolution of 0.25 views per degree in the high-demand views. An ultrawide viewing angle of 140° is also provided. The proposed light field 3D display does not require ultrahigh-resolution display panels and has form factors of thin and light. Thus, it has the potential to be used in portable electronics, window display, exhibition display, as well as tabletop display.

## 1 Introduction

Glasses-free three-dimensional (3D) displays have attracted great attention. The potential applications include intelligent manufacturing, telemedicine, automobile, and social networking [1–6]. Because of its thin form factor, light field 3D display is one of the glasses-free 3D display technologies that may re-define the information interaction in portable electronics [7–15]. Generally speaking, spatial and angular resolutions are both important parameters to evaluate the display performance in a 3D display system [7].

The angular resolution of a light field 3D display is defined as the view density, i.e. the number of views within the unit viewing angle.

(1) $$VD=\frac{1}{\Delta \theta }$$

where Δ*θ* is the angular separation of views. The units of angular resolution are views per degree (vpd). The spatial resolution is defined as pixel density with a unit of pixel per inch (ppi). The information density is further adopted to define the display information within the unit viewing angle:

(2) $$ID=\frac{N_{\text{mul}}}{\Delta \theta }$$

where *N*_mul_ is the pixel number of images at each view. The units of information density are pixels per degree (ppd).

Low spatial resolution may lead to clear pixelation effect, while low angular resolution causes discontinuous motion parallax. An ideal 3D virtual scene provides both high spatial and angular resolution for observers. Unfortunately, when total information is limited, there exists a fundamental trade-off between spatial resolution, angular resolution and field of view (FOV) (Figure S1).

Screen splicing strategy is an effective method to improve the total refreshable information, thus improving the 3D visual perception [16–18]. For example, four 8 K liquid crystal display (LCD) panels were combined to improve the display performance in an integral display [16]. The multi-screen splicing display prototype provided integral 3D images with a spatial resolution of 25 ppi within a viewing angle of 28°. The time-multiplexed scheme is another method to increase the amount of display information [19–22]. An angular steering screen was adopted to sequentially deflect light beams [19]. The prototype can produce smooth horizontal parallax with an angular resolution of 1 vpd within the horizontal viewing angle of ∼31°. Yet, increasing the information of display panel is bound to create stringent demands on panel fabrication, image rendering, circuit driving, system complexity and real-time data transport.

The human-centered mechanism inspired us with an alternative strategy to address the trade-off between spatial resolution, angular resolution and field of view [23, 24]. For example, the on-demand distribution is a human-centered strategy in the fields of sociology, economics and energy storage, which is used to handle the increasing number of requests against limited social resources [25–32]. Similarly, when the total information resource is limited, non-uniform distribution of information according to watching habit is preferable, although uniformly distributed information is taken as granted and widely adopted in all kinds of 3D displays.

Our group has proposed foveated glasses-free 3D display by creating space-variant view distribution via the 2D-metagrating complex [33]. A video-rate full color 3D display was demonstrated with an unprecedented 160° horizontal viewing angle and a spatial resolution of 52 ppi. The 2D-metagrating complex is designed by encoding the multiple periods and multiple orientations of the nanostructures in each pixel. The pixelated 2D metagrating modulates the phase of incident light for different output diffraction angles that generates the space-variant views [34, 35]. With the non-uniform angular sampling, the trade-off between angular resolution and FOV is solved. However, the low spatial resolution still needs to be improved.

In this paper, we further propose a light field 3D display with a non-uniform distribution of information and energy based on human watching habit of display screen. The rendered parallax images are non-uniformly projected to viewing regions through metagrating complex. For those frequently observed regions (called ‘high-demand view’), high spatial resolution images are projected to dot-shaped views with high angular resolution. For the less frequently observed region (called ‘low-demand view’), relatively low spatial resolution images are reconstructed in line-shaped views. Furthermore, the brightness of high-demand view and low-demand view is also distributed non-uniformly for the best usage of energy.

In the experiment, a large-scale metagrating complex (18 cm × 11.3 cm) with more than 12 million pixelated metagrating is fabricated. Integrated with an off-the-shelf purchased display panel (total pixel number: 2560 × 1600), a video-rate full color light field 3D display is demonstrated. The spatial resolution is increased up to 119.6 ppi and the angular resolution is enhanced up to 0.25 vpd at the high-demand views. The system also provides an ultrawide viewing angle of 140°. The proposed system requires neither ultra-high-resolution display panels nor time-/spatial-multiplexing architectures, thus it features the potential applications in portable electronic devices by integrating with commercially available display panels.

## 2 Light field 3D display with space-variant resolution

**Figure 1:**
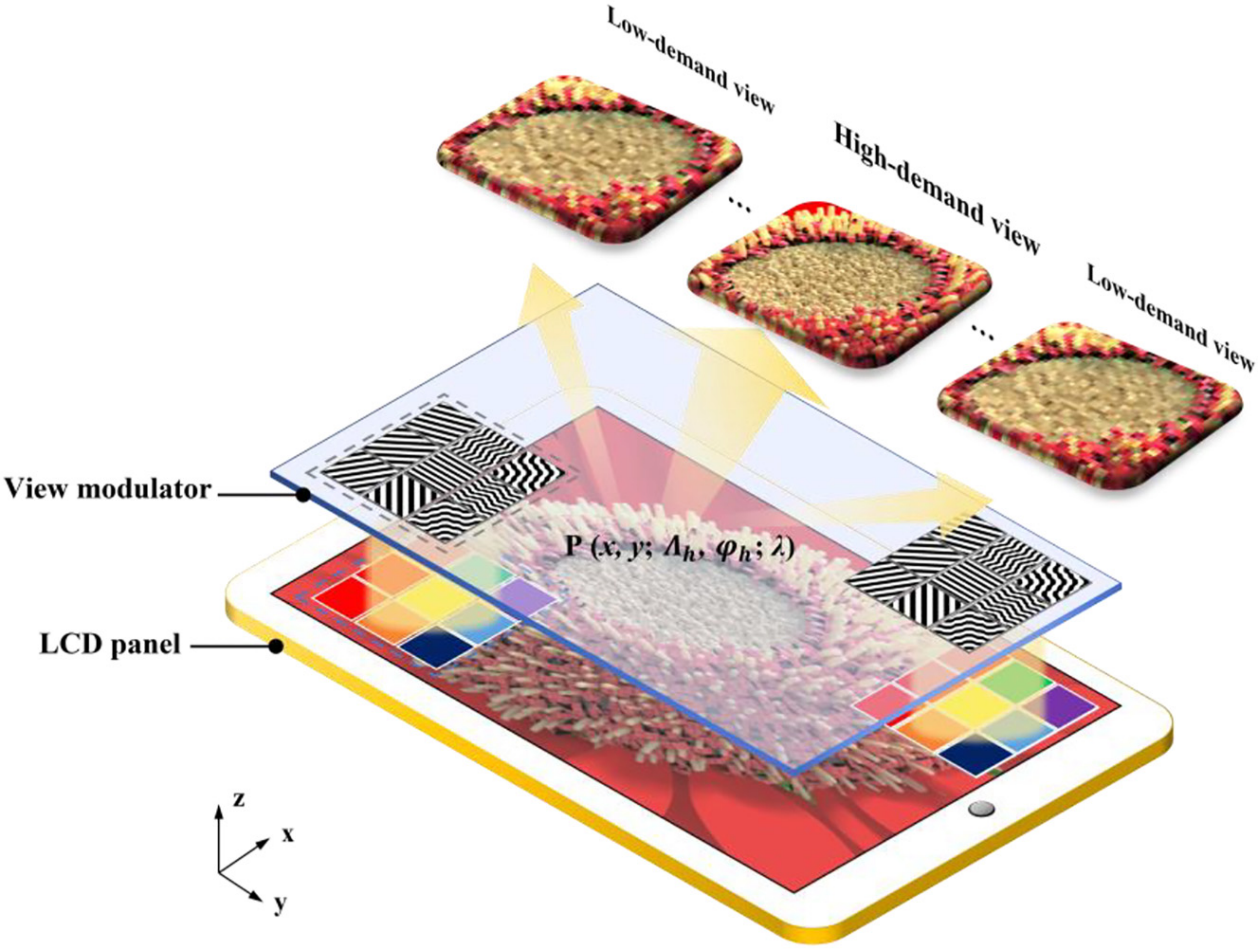
Schematic diagram of the proposed light field 3D display with space-variant resolution.

Figure 1 illustrates the system configuration of the proposed light field 3D display. The prototype has a compact form factor and is composed of a view modulator and a display panel. For illustration purpose, a voxel on an LCD panel contains 3 × 3 pixels. In the traditional strategy, each pixel in a voxel is usually directed to one view [12, 36]. As a result, 9 views with uniformly distributed spatial and angular resolution are rendered. Here we redistribute each pixel so that the spatial resolution of 3D image at the high-demand view is increased. With the space-variant pixel numbers, the spatial resolution of the display prototype is redistributed non-uniformly.

Moreover, the space-variant angular resolution is enabled by the view modulator. The view modulator covered with metagrating complex is aligned with the LCD panel pixel by pixel. The metagrating complex is designed to tailor the output diffraction angles. The angular divergence of diffractive beam in horizontal direction spans from 1° to 10°. As a result, when a collimated white light illuminates on the prototype, the light field 3D display with space-variant resolution is achieved. The strategy of space-variant resolution seems straightforward but is nontrivial. It should be noted that the light field from each pixel need to be manipulated independently. Light modulation at pixel level can hardly be achieved by traditional geometric optics, such as lenticular lens, or microlens.

### 2.1 Modulation method for space-variant spatial resolution

**Figure 2:**
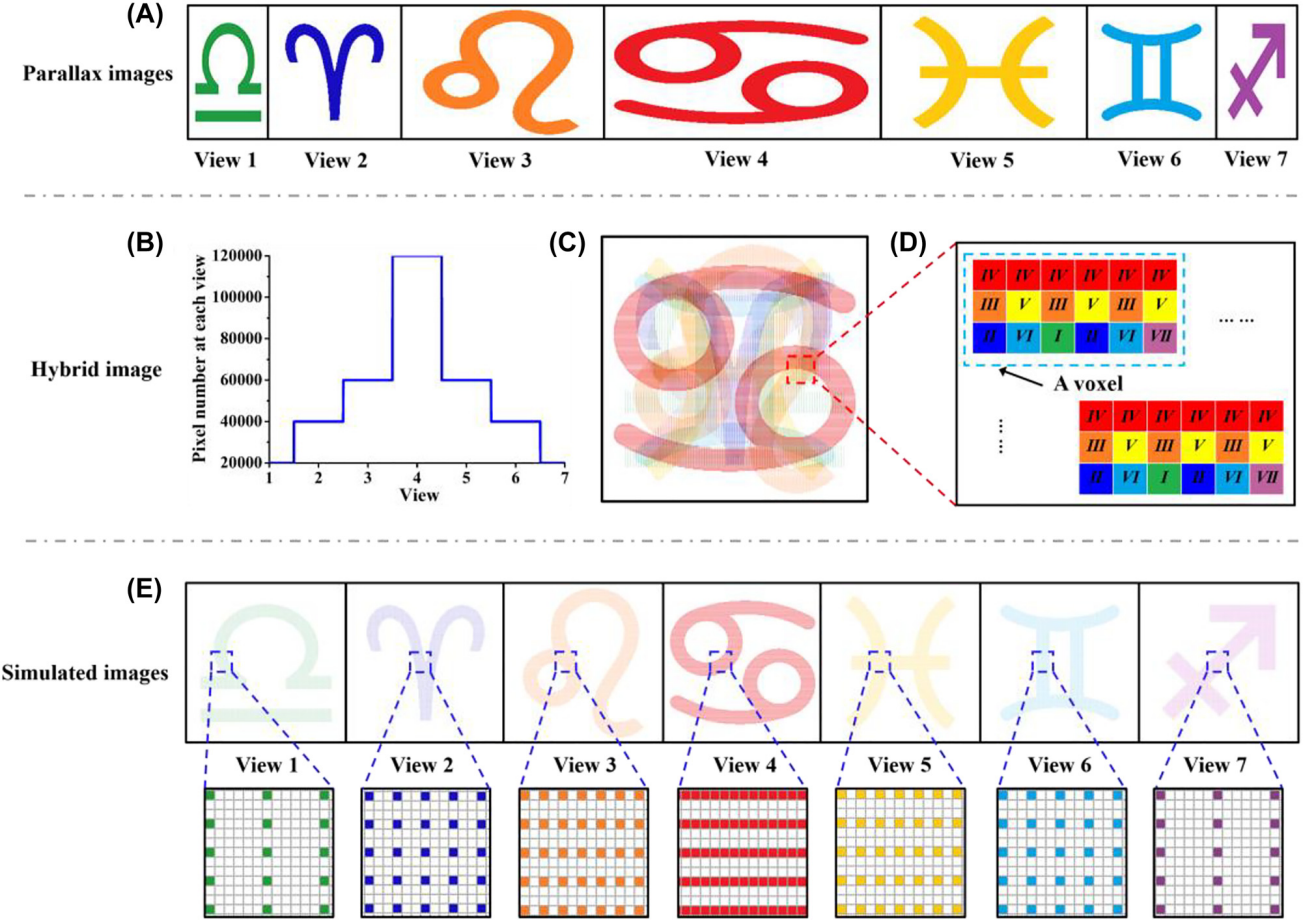
Schematic of the modulation method of spatial resolution in the horizontal direction. (A) Seven ‘astrological symbol’ parallax images with various pixel number in the horizontal direction. The pixel number is 100 × 200 at View 1 & 7, 200 × 200 at View 2 & 6, 300 × 200 at View 3 & 5 and 600 × 200 at View 4, respectively. (B) Distribution of pixel number at each view. (C) The hybrid image which contains seven parallax images. (D) Schematic of the arrangement of pixels in the voxels. There are 6 × 3 pixels in one voxel. (E) The simulated images observed at each view.

Generally speaking, a multi-view camera system or a 3D computer graphic software is used to capture 2D images from different perspectives. We refer to these individual perspective images as parallax images. As shown in Figure 2A, seven ‘astrological symbol’ parallax images are captured at seven views, respectively. The pixel number is decreased from 600 × 200 at View 4 to 100 × 200 at View 1, 7. A Gaussian-like distribution of pixel number along views provide significantly increased spatial resolution at high-demand view (Figure 2B).

Then the hybrid image is created by merging a series of parallax images, as shown in Figure 2C. The total pixel number of hybrid image is 600 × 600. In traditional voxel arrangement, each voxel contains 7 pixels with one pixel from each view. In contrast, one pixel from View 1, 7, two pixels from View 2, 6, three pixels from View 3, 5, and six pixels from View 4 attribute to a voxel (Figure 2D). As a result, a voxel consists of 6 × 3 pixels and the total voxel number is (600 ÷ 6) × (600 ÷ 3) = 100 × 200. Therefore, the spatial resolution of high-demand view is increased to 171 ppi at View 4, while the spatial resolutions of low-demand views are decreased to 97 ppi at View 3, 5, 76 ppi at View 2, 6 and 60 ppi at View 1, 7 (Figure 2E). With the non-uniform pixel arrangement, the spatial resolution and the brightness are variant in space. Moreover, other possible strategies of spatial resolution modulation are provided in Section S2 in the Supplementary material.

### 2.2 Modulation method for space-variant angular resolution

The general grating equation for an arbitrary obliquely incident beam can be written in direction cosine space based on the Raman–Nath regime [37].

(3) $$n\alpha_{i}-\alpha_{m}=\left(m\lambda /\Lambda_{\text{h}}\right)\sin\varphi_{h}$$

(4) $$n\beta_{i}-\beta_{m}=\left(m\lambda /\Lambda_{h}\right)\cos\varphi_{h}$$

where *α*_*i*_, *β*_*i*_, *α*_*m,*_ and *β*_*m*_ are the direction cosines of the incident beam and diffraction beam, respectively, *λ* is the wavelength of beam, *m* is the diffraction order, *n* is the refraction index, Λ_*h*_ and *φ*_*h*_ are the multiple periods and multiple orientations of the metagrating, respectively, and *h* is the period serial number in the metagrating pixel (*h* = 1,2,3 …). Combining Eq. (3) and Eq. (4), the structural parameters of metagratings (Λ_*h*_ and *φ*_*h*_) are calculated pixel by pixel [33, 35]. Therefore, the metagrating complex controls the emitting light direction at pixel-level with a high precision, which can be expressed as follows [38, 39]:

(5) $$U=P\left(x,y;\Lambda_{h},\varphi_{h};\lambda \right)$$

where (*x*, *y*) are the coordinate values of the pixel on the view modulator plane. 1D metagratings converge the emitting light from the LCD panel to form dot shaped views in the central region. 2D metagratings reconstruct the phase of the emitting beam to form horizontal line shaped views in the periphery region. With the non-uniform view distribution, the angular resolution is modulated to be variant in the space.

**Figure 3:**
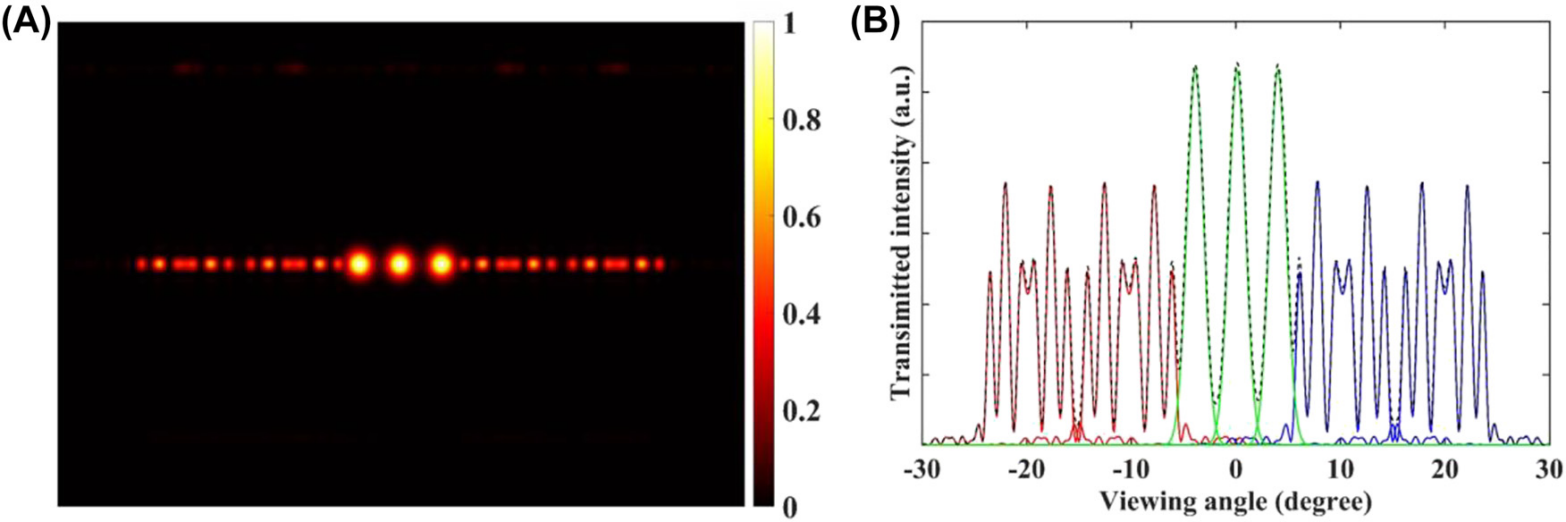
The finite-difference time-domain (FDTD) simulation of the metagrating complex. (A) The 2D radiation pattern from the metagrating complex. The dot shaped views are distributed in the center region, while the line shaped views are arranged on both edges. (B) The intensity distribution along the horizontal direction.

In the proposed prototype, the angular separation of dot shaped views (high-demand views) and line shaped views (low-demand views) are set as 4° and 10°, respectively. Therefore, the angular resolution of the high-demand view is at most 2.5 times that of low-demand views. Figure 3A and B show the simulated radiation pattern and intensity distribution along the horizontal direction. The simulated diffraction efficiencies are 15.5% and 6.6% for 1D metagratings and 2D metagratings (*λ* = 530 nm) with a depth of 200 nm. The crosstalk of the central view is around 2%.

## 3 Design and fabrication of metagrating complex

**Figure 4:**
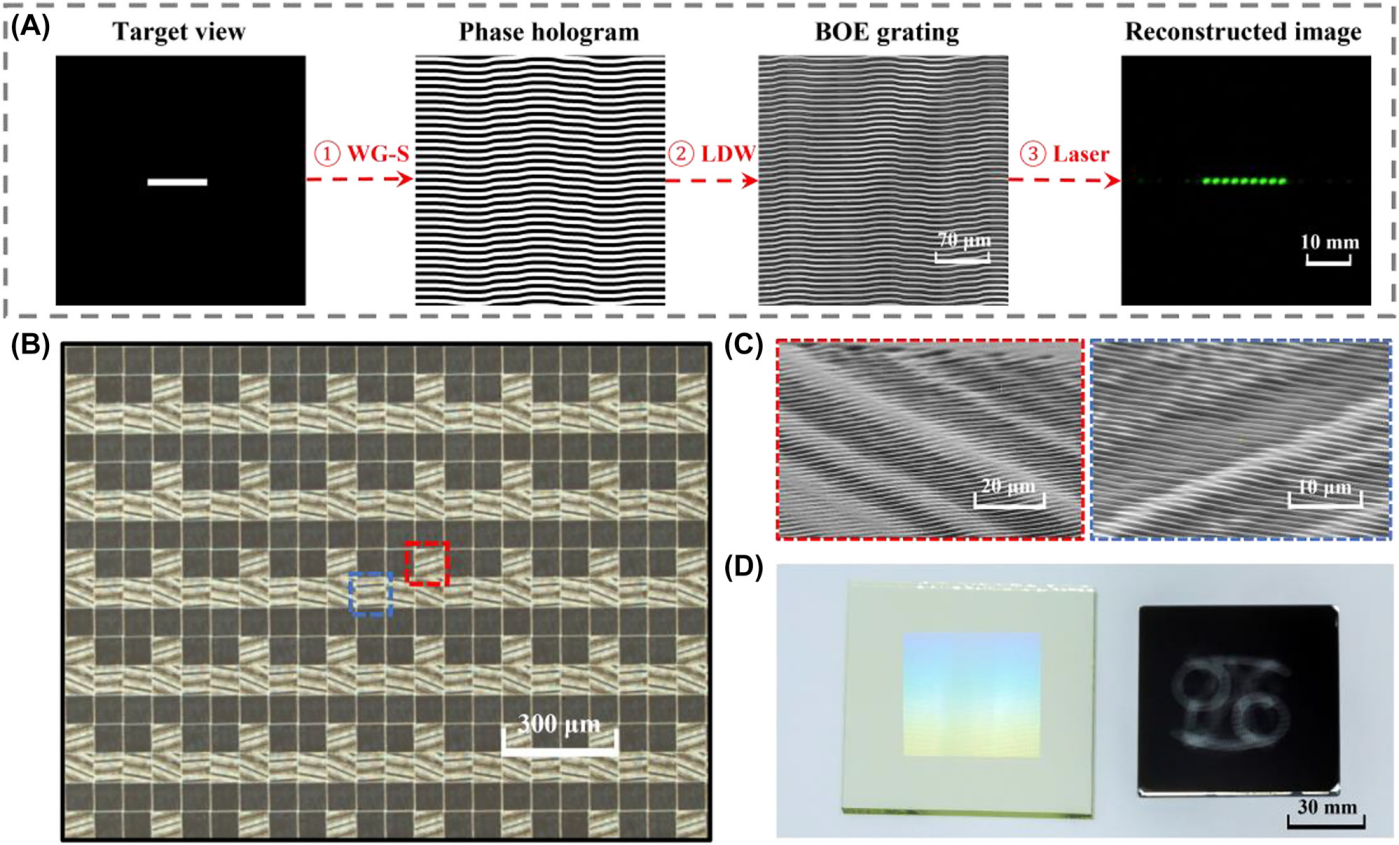
Design and fabrication flow of the metagrating complex: (A) design process of the BOE grating: ① generating the phase hologram using a weighted G-S algorithm; ② transforming the phase hologram into the BOE using laser direct writing; ③ reconstructing the target image using a 532 nm laser. (B) The microscope image of the pixelated metagrating complex. (C) Magnified SEM images of 2D metagratings. (D) Photo of the fabricated view modulator and a shadow mask.

The pixelated metagrating complex plays a crucial role in the proposed display system to form the non-uniform distribution of information density and energy. A home-made light field direct writing system is built to pattern metagratings pixel by pixel [40, 41]. Figure 4 shows the design and fabrication process of the metagrating complex. Firstly, we adopt a weighted G-S algorithm to generate the phase hologram based on the target view [42, 43]. Then the phase hologram is transformed into a BOE grating on glass substrate after laser direct writing (LDW) and dry etching process. The reconstructed image of the fabricated BOE grating has line shaped irradiance under the illumination of a 532 nm laser (Figure 4A). The prepared BOE grating is inserted into the light field direct writing system. By moving and rotating the inserted BOE grating between two Fourier transform lens within the system, metagratings are fabricated with predesigned multiple periods and orientations [44]. We take the microscopic image and magnified SEM images of metagratings, respectively (Figure 4B and C). To prove the concept of the light field 3D display, a 3.7 inch view modulator and a shadow mask are fabricated, as shown in Figure 4D. The view modulator contains 200 × 200 voxels, and each voxel has 3 × 3 pixelated metagratings.

**Figure 5:**
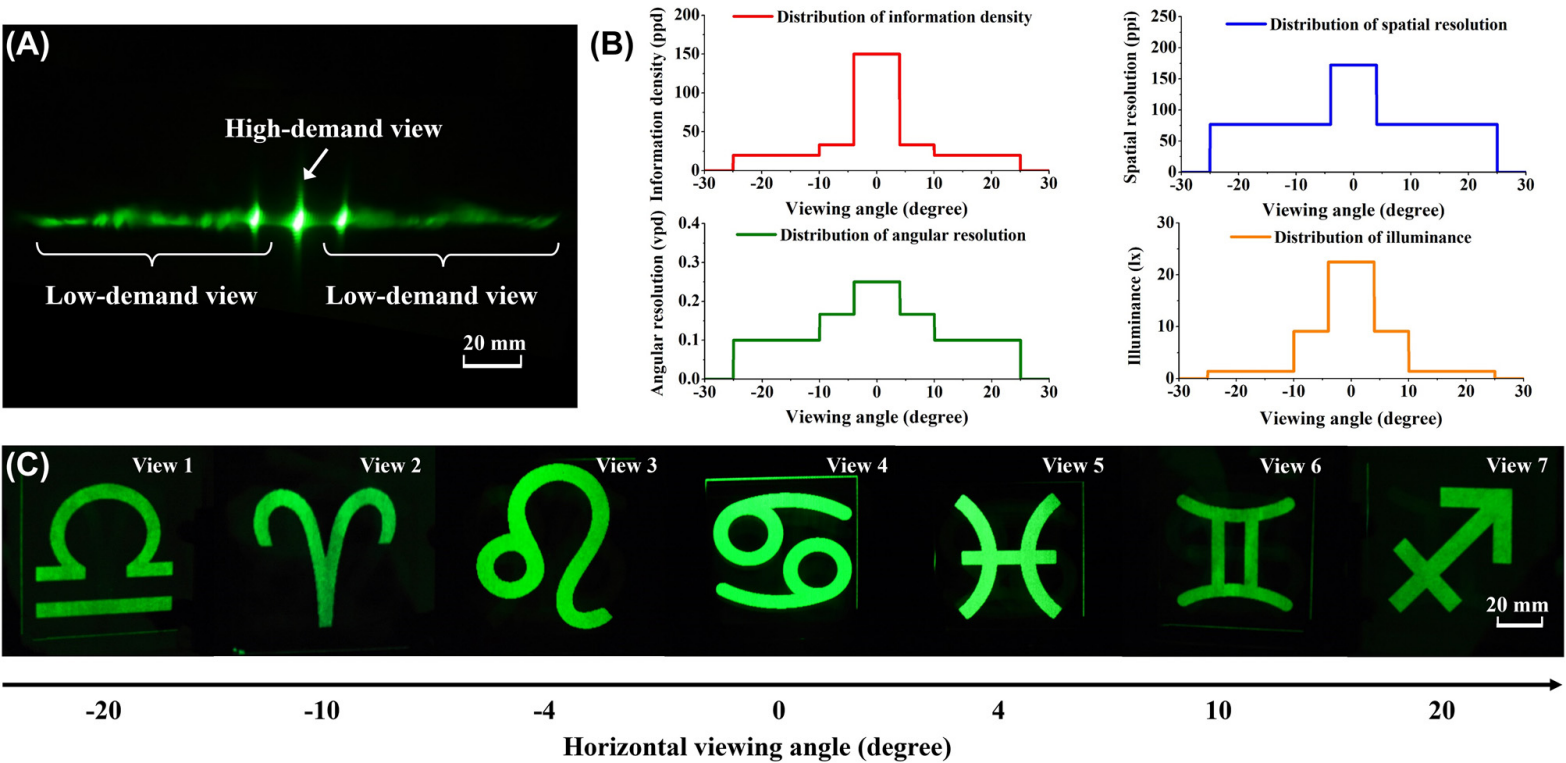
A monochromatic light field 3D display prototype with space-variant resolution: (A) the measured radiation pattern of the view modulator. (B) The distribution of spatial resolution, angular resolution, information density, and illuminance, respectively. (C) 3D images of astrological symbols observed from View 1–7.

Figure 5A presents the measured radiation pattern of the view modulator, under the illumination of a 532 nm laser source. Three dot-shaped views are distributed in the central region with an angular resolution of 0.25 vpd. The central views are sandwiched by four line-shaped views with an angular resolution of 0.1 vpd. Besides, the angular resolution of the region between dot-shaped views and line-shaped views is 0.17 vpd. The spatial resolution of the high-demand view is 2.2 times more than the spatial resolution of low-demand views. The typical parameters of the 3.7 inch view modulator are listed in Table 1. Figure 5B illustrates the distribution of spatial resolution, angular resolution, information density, and illuminance, respectively. With the joint modulation of pixel density and view arrangement, the information density of the high-demand view is increased to at most 5.6 times that of low-demand views.

**Table 1:** Optical performance of three typical prototypes.

3D Imaging characteristics	View Modulator in Figure 5	View Modulator in Figure 6	View Modulator in Figure 8
Frame size	3.7 inch	5.1 inch	8.4 inch
View number	7	24	24
Number of pixels	600 × 600	5400 × 1200	2560 × 1600
Field of view	50°	140°	140°
Spatial resolution^a^	170.9 ppi (H)	361.6 ppi (H)	119.6 ppi (H)
	76.4 ppi (L)	180.8 ppi (L)	59.8 ppi (L)
Angular resolution	0.25 vpd (H)	0.25 vpd (H)	0.25 vpd (H)
	0.17 vpd (L)	0.2 vpd (L)	0.2 vpd (L)
	0.1 vpd (L)	0.1 vpd (L)	0.1 vpd (L)
Information density	158.1 ppd (H)	461 ppd (H)	251.1 ppd (H)
	47.1 ppd (L)	184.4 ppd (L)	100.4 ppd(L)
	28.3 ppd (L)	92.2 ppd (L)	50.2 ppd (L)

^a^H, high-demand view; L, low-demand view.

The space-variant information density is first tested by a monochromatic 3D image. A view modulator and a shadow mask are aligned pixel by pixel. Under the illumination of a green light-emitting diode (LED) light, seven astrological symbols are projected to each view, as shown in Figure 5C. The prospective images are well separated from the high-demand view and low-demand views (Visualization 1). The illuminance is further measured at each view. The illuminance decreases from 22.5 lx at View 4 to 1.4 lx at View 1, 2, 6, 7. Energy redistribution provides a strategy to minimize optical power consumption.

## 4 Characteristics of display prototype

**Figure 6:**
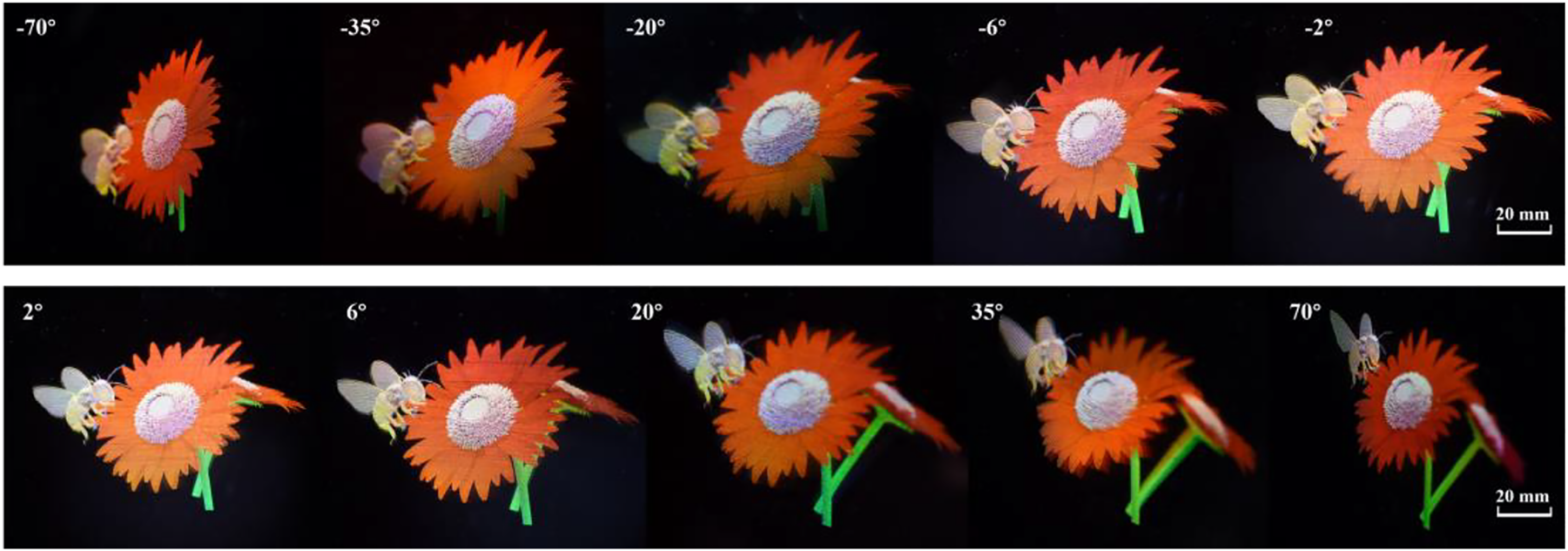
3D images of ‘bees and flowers’ observed from various views with natural motion parallax and color mixing.

To further validate the light field 3D display with space-variant resolution, multiple full-color view modulators with different frame sizes are fabricated. A full color light field 3D display is demonstrated by integrating a 5.1 inch view modulator and a shadow mask. Figure 6 shows the 3D images of ‘bees and flowers’ which are reassigned and redirected to various views. The spatial resolution of high-demand views (−6°, −2°, 2°, and 6°) are 2 times that of other views.

**Figure 7:**
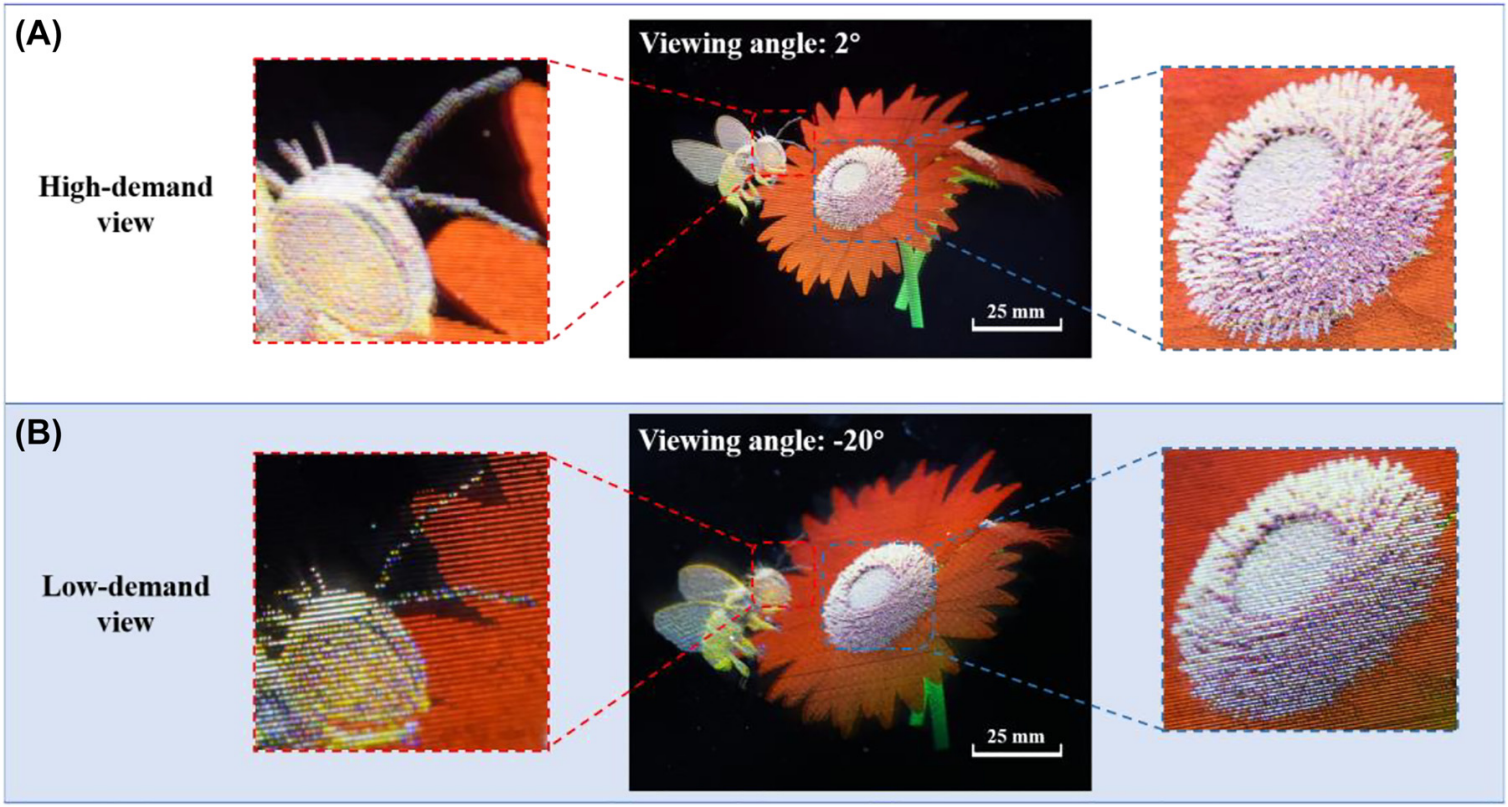
A comparison between the magnified photos taken at (A) the high-demand view, and (B) the low-demand view.

We take a comparison between the high-demand view and low-demand view, as shown in Figure 7. The magnified images (bee’s head and pistil) at the high-demand view can express the details clearly, while the magnified images at the low-demand view become blurred due to the pixelation effect. The information density of high-demand views is increased up to 461 ppd, which is at most 5 times that of low-demand views.

**Figure 8:**
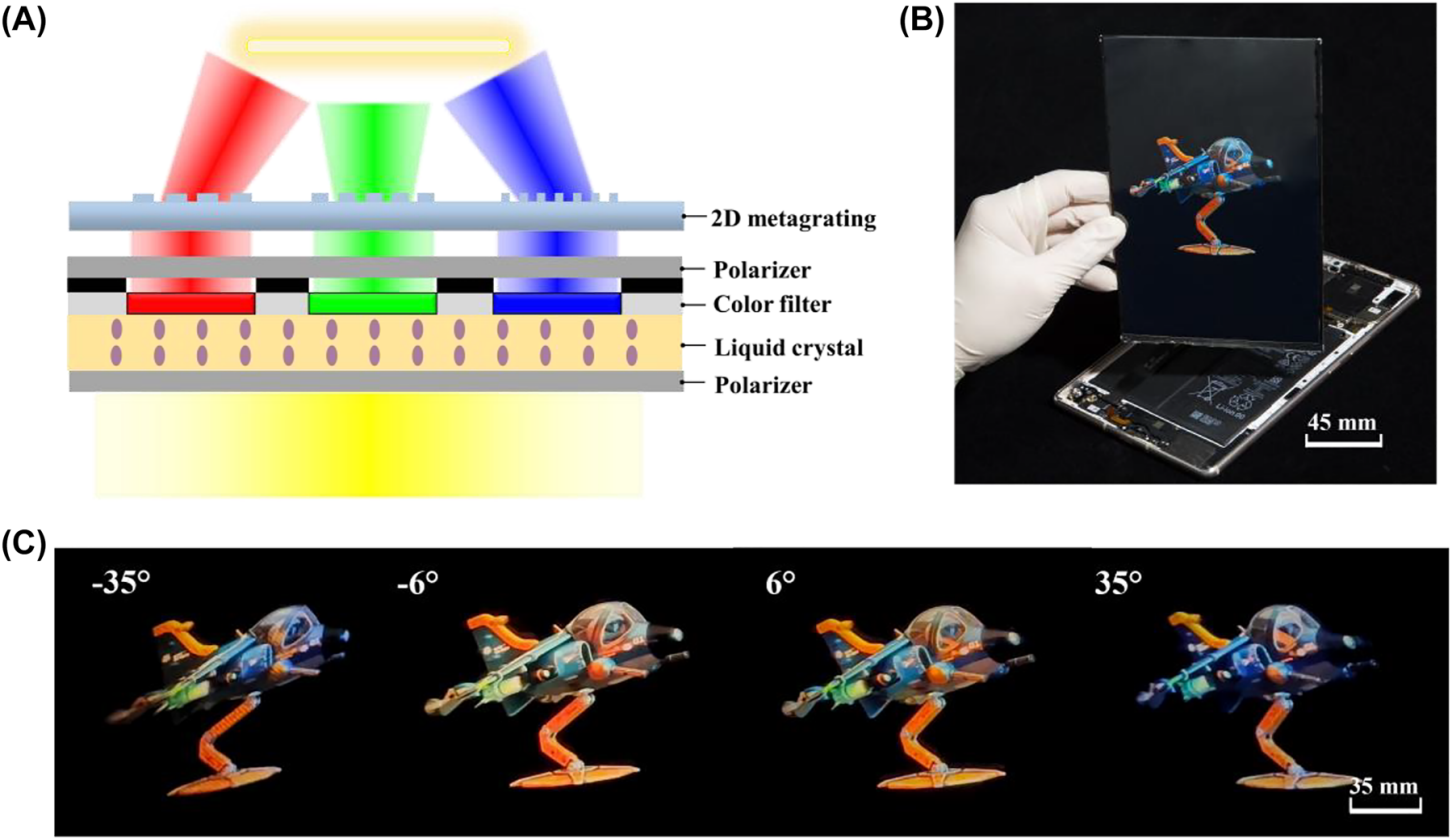
A full-color and video rate light field 3D display with space-variant resolution: (A) schematic of a combined pixel in the full color 3D display prototype. (B) A demo of the 8.4 inch light field 3D display. (C) 3D images of ‘cartoon plane’ observed from left to right views.

Furthermore, by integrating the view modulator with an 8.4 inch off-the-shelf LCD panel (M6, HUAWEI), a video rate and full color light field 3D display prototype is demonstrated. The typical parameters of the full color prototypes are listed in Table 1. Figure 8A depicts the individually modulated R/G/B subpixels for full color display, by stacking 2D metagratings, polarizers, color filters, and liquid crystals, successively. The prototype is achieved by aligning the view modulator and the LCD panel pixel by pixel via one-step bonding assembly (Figure 8B).

We capture images with a camera (D810, Nikon) at a viewing distance of 500 mm (Figure 8C and Figure S6). The proposed light field 3D display features high spatial resolution of 119.6 ppi and high angular resolution of 0.25 vpd at high-demand views. The viewing angle with continuous motion parallax is 140° (Visualization 2 & 3). The information density at high-demand views is increased up to 251.1 ppd.

**Figure 9:**
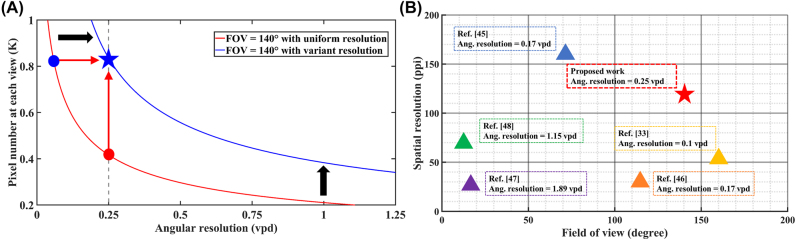
The proposed 3D display with variant information distribution and other 3D displays: (A) Schematic of the relationship between spatial resolution and angular resolution in two different modes. The blue star represents the parameters of the 8.4 inch light field 3D display prototype. The pixel number and angular resolution are 0.83 K (852 × 532) and 0.25 vpd at the high-demand views, respectively. (B) Comparison of the display performance between the proposed non-uniform strategy and the state-of-art 3D displays.

Finally, we take a comparison between the conventional 3D display with uniform information distribution and the proposed 3D display with variant information distribution, as shown in Figure 9A. For a fair comparison, the total display information remains unchanged and set as 2560 × 1600. Benefiting from the non-uniform distribution strategy, the trade-off curve dramatically shifts right. To be specific, when the FOV is set as 140° and the angular resolution reaches 0.25 vpd, the pixel number is increased from 0.42 K to 0.83 K (From the red dot to the blue star). If we keep the pixel number at 0.83 K, the angular resolution is increased from 0.06 vpd to 0.25 vpd (From the blue dot to the blue star). The experimental results marked by the blue star demonstrate the feasibility of the method. We further compare the proposed work with prior art [33, 45], [46], [47], [48]. As shown in Figure 9B, the viewing angle is greatly expanded while maintaining a high spatial resolution and angular resolution at the high-demand views.

## 5 Discussion and conclusion

Based on the watching habits in the flat panel display, high-demand views are placed at the central region in current prototype. Yet the high-demand views can be placed in other areas. For example, in a vehicle dial display system, high-demand views are better concentrated in the periphery region. In a near-eye display, high-demand views should be shifted to where the eye gaze reaches.

Here we sandwiched high-demand views with low-demand views along the horizontal direction for horizontal parallax. Alternatively, vertical parallax and full parallax can be achieved by a group of dot/line/rectangle-shaped views [33].

To summarize, a facile and effective strategy for the light field 3D display with space-variant resolution was demonstrated. Non-uniform distribution of information and energy is achieved according to human watching habit. The pixel density of parallax images on various views is adjusted to achieve the variant spatial resolution. Meanwhile, the brightness of the parallax images also forms a non-uniform distribution. The light consumption at the low-demand views can be saved which makes the proposed light field 3D display energy efficient.

Using a homemade versatile light field direct writing system, multiple view modulators composed of 1D metagratings and 2D metagratings were fabricated with a frame size ranging from 3.7 inch to 8.4 inch. More than 12 million pixels of metagratings were patterned. Seven dot and line hybrid views with horizontal distribution were achieved. With the joint modulation of pixel density and view distribution, the information density and illuminance at the high-demand views were increased to at most 5.6 times and 16 times that of low-demand views.

Moreover, by integrating the view modulator with an 8.4 inch off-the-shelf purchased LCD panel, a video-rate and full-color display prototype was built. With total display information of 2560 × 1600, the prototype achieved a high spatial resolution of 119.6 ppi and a high angular resolution of 0.25 vpd at high-demand views. The prototype also provided an ultrawide motion parallax within 140°. In theory, the maximum viewing angle can reach 180° by properly designing the nanostructures. However, people seldom observe a display panel at an angle of ±90°. A FOV of ∼140° is adequate for portable electronics in most cases.

One should note that metagrating complex is not the only pattern that can achieve non-uniform information distribution. More complexed nanostructures [49] can be adopted to further improve the display performance in terms of FOV, light efficiency or depth of viewing distance. Yet the design and fabrication of sophisticated nanostructures over a function display size (larger than 4 inch) is a critical issue that hinders the advancement of nanophotonics based glasses-free 3D display.

The proposed light field 3D display featured thin and light form factors. We expect that it can be potentially applied in consumer electronic devices. Furthermore, future studies in the directional backlight module equipped with eye tracking system may open new avenues for its integration and commercialization.

## 6 Methods

### 6.1 Simulation section

The numerical simulations were completed in the FDTD software (FDTD solutions, Ansys/Lumerical). The 3D structure data of metagrating complex were imported into the FDTD as the form of GDSII (By LinkCAD). The refractive index of the photoresist was set as 1.62 (*λ* = 530 nm). The groove depth of structures was set as 200 nm. The pixel size was set as 30 µm. Two plane wave sources, which were served as natural light, illuminated on the metagrating complex with an incident angle of 30°. The boundary conditions were set as Bloch and perfect matching layer (PML) along transverse and longitudinal directions, respectively. The radiation pattern was produced in the far field projection of the monitors.

### 6.2 Fabrication section

(1) For the inserted BOE gratings: First, we pre-cleaned a 2.5 inch quartz plate. The quartz substrate was then coated with HMDS (DisChem, SurPass 3000) and positive photoresist (AZ^®^ P4620, MicroChemicals) sequentially. Second, the quartz plate was patterned with binary phase hologram using LDW system (MiScan, SVG optronics). The BOE gratings structure were fabricated through the photolithography and developed in NaOH solution. The concentration of NaOH solution was about 8‰. Third, to increase the photoresist durability, the BOE gratings were etched using the plasma etching system (NLD-570, ULVAC). As a result, the groove depth was approximately 600 nm and the average period was 7.5 µm. Finally, the BOE gratings were inserted in the versatile interference lithography to fabricate the metagrating complex.
(2) For the view modulators: First, we pre-cleaned a glass plate. The size of glass plate was determined by the display frame size. The glass plate was then coated with HMDS and positive photoresist (RJZ-390, RUIHONG Electronics Chemicals). Second, 1D metagratings and 2D metagratings were patterned by inserting the BOE grating into the interference lithography, respectively. Third, the view modulator was aligned with the LCD panel or shadow mask pixel by pixel. The alignment process was conducted using four high-resolution CCD microscopes (HG-3101BT, MHAGO).

## Supplementary Material

Supplementary Material Details

## Supplementary Material

The article contains supplementary material (https://doi.org/10.1515/nanoph-2022-0637).
