# Supplementary material for: Large-scale metagrating complex-based light field 3D display with space-variant resolution for non-uniform distribution of information and energy
Source: Nanophotonics. 2023 Jan 13;12(2):285–95. doi: 10.1515/nanoph-2022-0637 (PMC11501163; doi:10.1515/nanoph-2022-0637)
Supplement: Supplementary file 1 — Supplementary Material Details [file j_nanoph-2022-0637_suppl.doc]

Supplementary Material

Title Large-scale metagrating complex-based light field 3D display with space-variant resolution for non-uniform distribution of information and energy

Jianyu Hua, Fengbin Zhou, Zhongwen Xia, Wen Qiao*, Linsen Chen*

*Corresponding authors: **Wen Qiao**, School of Optoelectronic Science and Engineering & Collaborative Innovation Center of Suzhou Nano Science and Technology, Soochow University, Suzhou 215006, China; and Key Lab of Advanced Optical Manufacturing Technologies of Jiangsu Province & Key Lab of Modern Optical Technologies of Education Ministry of China, Soochow University, Suzhou 215006, China, e-mail: wqiao@suda.edu.cn

**Linsen Chen**, School of Optoelectronic Science and Engineering & Collaborative Innovation Center of Suzhou Nano Science and Technology, Soochow University, Suzhou 215006, China; and Key Lab of Advanced Optical Manufacturing Technologies of Jiangsu Province & Key Lab of Modern Optical Technologies of Education Ministry of China, Soochow University, Suzhou 215006, China; and SVG Optronics, Co., Ltd, Suzhou 215026, China, e-mail: lschen@suda.edu.cn

**Jianyu Hua**, **Fengbin Zhou** and **Zhongwen Xia**: School of Optoelectronic Science and Engineering & Collaborative Innovation Center of Suzhou Nano Science and Technology, Soochow University, Suzhou 215006, China; and Key Lab of Advanced Optical Manufacturing Technologies of Jiangsu Province & Key Lab of Modern Optical Technologies of Education Ministry of China, Soochow University, Suzhou 215006, China

**Section S1: A supplementary to the trade-off in light field 3D display**

**Figure S1** shows the trade-off between spatial resolution, angular resolution and FOV when the total pixels of screen are limited. The pixel number on a screen is set to 2560×1600 (as same as the display panel in the experiment). The angular resolution decreases as the pixel number at each view increases. The curves shifts to the left when the FOV is enlarged. For example, when the FOV is 140° and the angular resolution reaches 0.25 vpd, the pixel number at each view is ~0.42 K (The red dot). But if we increase the pixel number to 0.83 K, the angular resolution decreases along the red curve to ~0.06 vpd (The blue dot). Therefore, the constraints between spatial and angular resolution limit the 3D visual perception.





Figure S1: Schematic of the trade-off between spatial resolution, angular resolution and FOV. Here, the spatial resolution is represented by the pixel number at each view. Assume that 1 K is 1024×640, so 0.5 K is (0.5×1024) × (0.5×640).

**Section S2: A supplementary to the modulation method for variant spatial resolution**

In order to increase the spatial resolution of the high-demand view vertically, a modulation method is given as follow. As shown in **Figure S2**, seven ‘astrological symbol’ parallax images are captured at seven views, respectively. The pixel number is decreased from 200 × 600 at View 4 to 200 × 200 at other views. The spatial resolution of high-demand view is increased siginificantly by the Gaussian-like distribution of pixel number (**Figure S3A**). **Figure S3B** shows the created hybrid image by merging the parallax images. The total pixel number of the hybrid image is 600 × 600. In this architecture, one pixel from View 1, 2, 3, 5, 6, 7 and three pixels from View 4 attribute to a voxel (**Figure S3C**). As a result, each voxel consists of 3 × 3 pixels and the total voxel number is (600÷3) × (600÷3) = 200 × 200. The spatial resolution of high-demand view is increased to 171 ppi at View 4, while the spatial resolutions of low-demand views are 76 ppi at View 1, 2, 3, 5, 6, 7 (**Figure S3D**).

To further increase the spatial resolution of the high-demand view both horizontally and vertically, a modulation method is also given as follow. As shown in **Figure S4**, seven ‘astrological symbol’ parallax images are captured. The pixel number is decreased from 600 × 300 at View 4 to 300 × 100 at other views. The spatial resolution of high-demand view is further improved by redistributing the pixel number (**Figure S5A**). **Figure S5B** shows the hybrid image which has a pixel number of 600 × 600. In this architecture, two pixels from View 1, 2, 3, 5, 6, 7 and eighteen pixels from View 4 attribute to a voxel (**Figure S5C**). As a result, a voxel consists of 6 × 6 pixels and the total voxel number is (600÷6) × (600÷6) = 100 × 100. The spatial resolution of high-demand view is increased to 181 ppi at View 4, while the spatial resolutions of low-demand views are 85 ppi at View 1, 2, 3, 5, 6, 7 (**Figure S5D**).


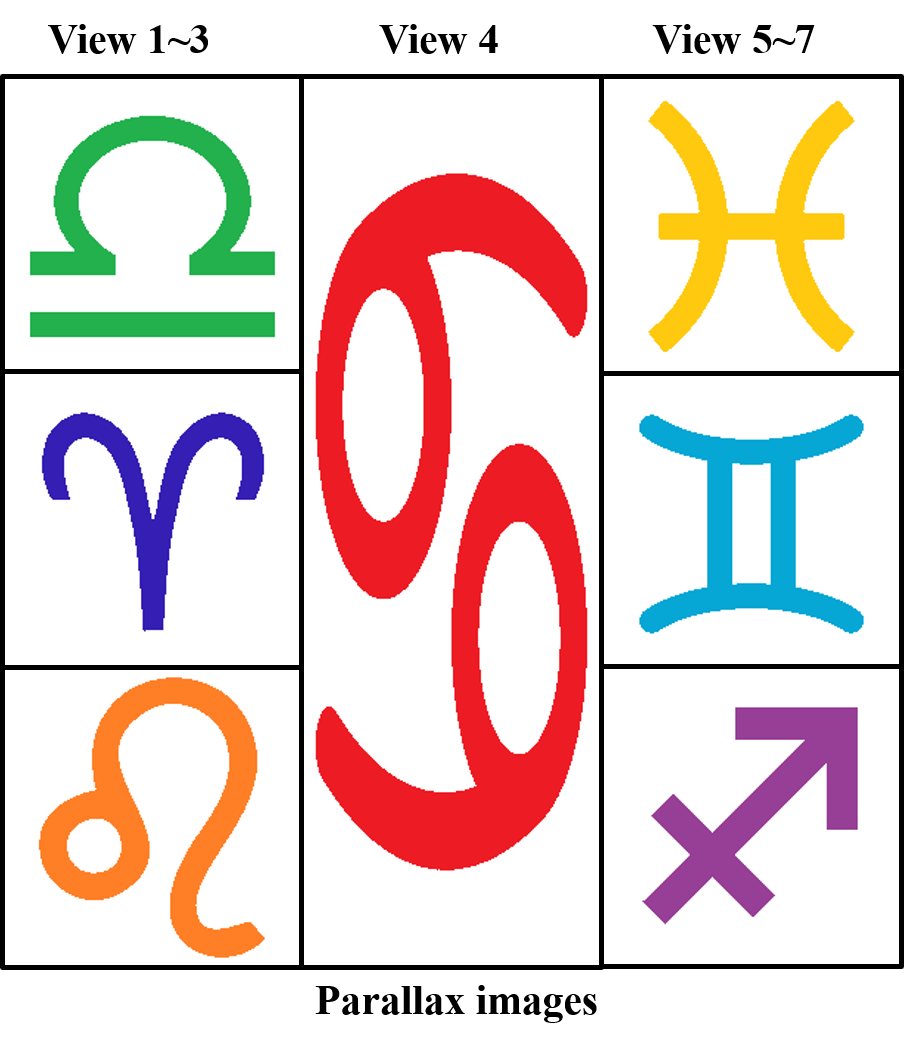


Figure S2. Schematic of seven ‘astrological symbol’ parallax images with space-variant pixel number in the vertical direction. The pixel number is 200 × 600 at View 4, 200 × 200 at View 1, 2, 3, 5, 6, 7.


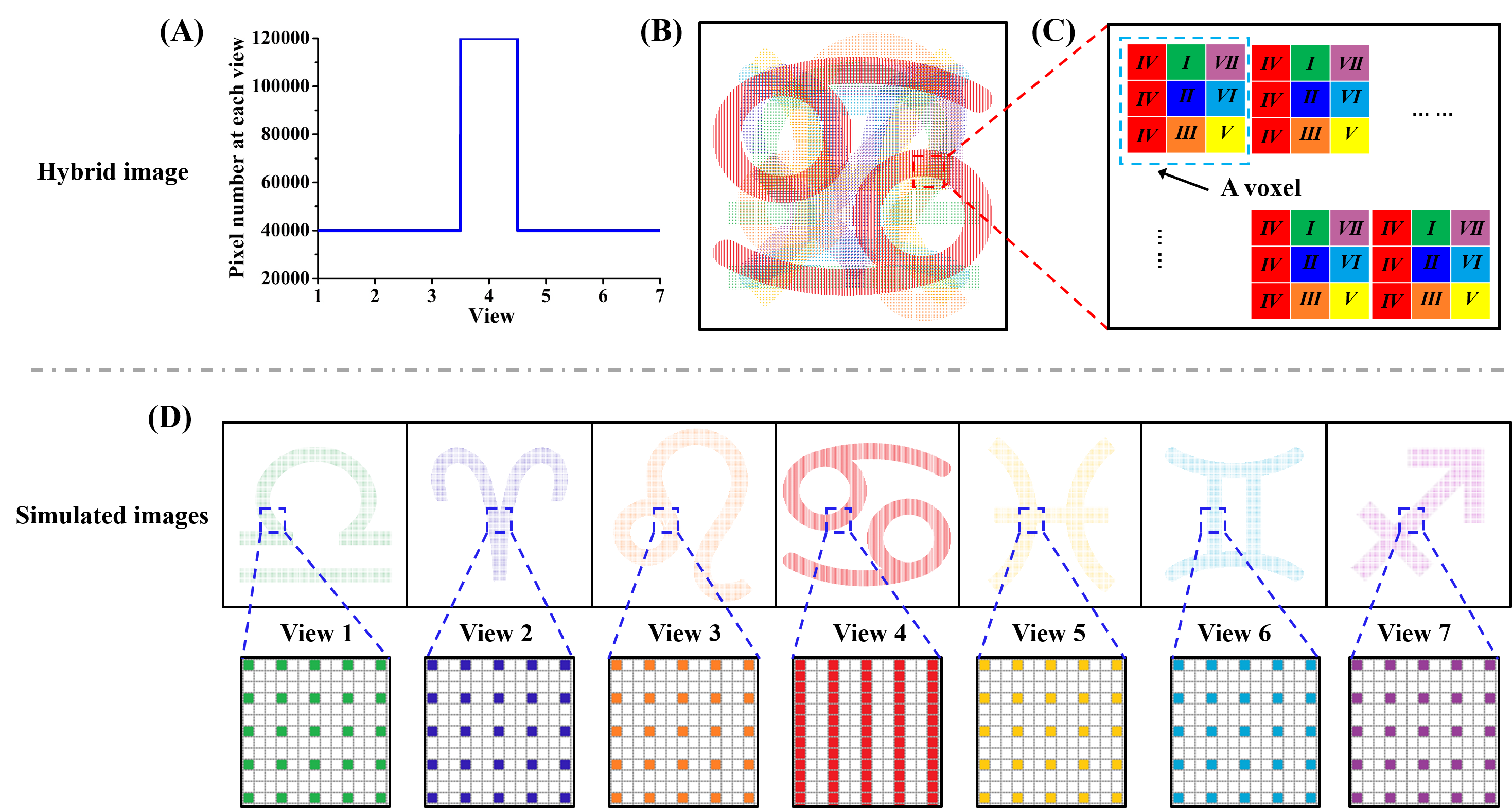


Figure S3. Schematic of the modulation method of spatial resolution in the vertical direction. (A) distribution of pixel number at each view. (B) the hybrid image which contains seven parallax images. (C) the arrangement of pixels in the voxels. There are 3 × 3 pixels in one voxel. (D) the simulated images observed at each view.

**
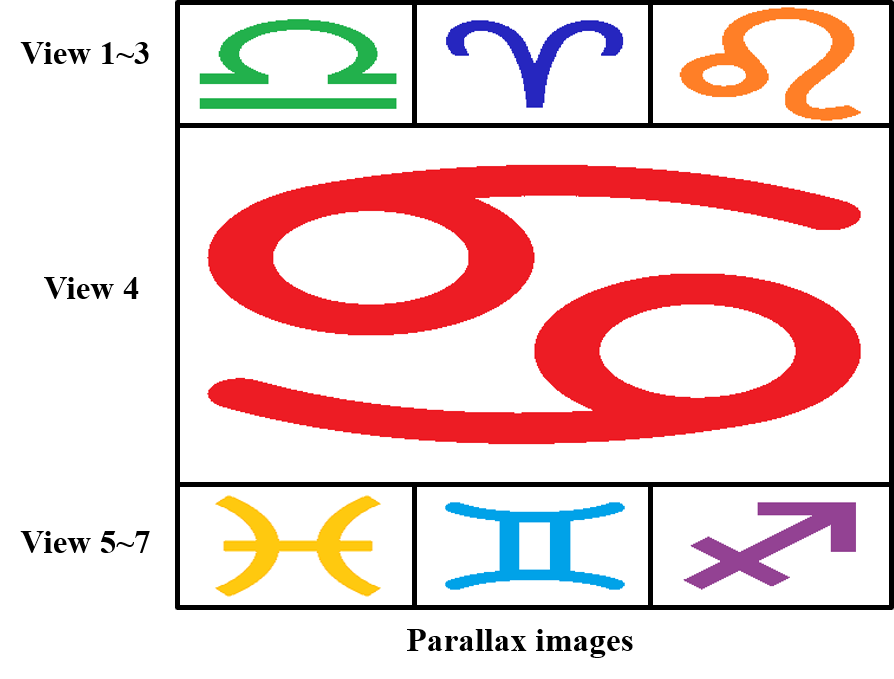
**

Figure S4. Schematic of seven ‘astrological symbol’ parallax images with space-variant pixel number both in the horizontal and vertical direction. The pixel number is 600 × 300 at View 4, 300 × 100 at View 1, 2, 3, 5, 6, 7.


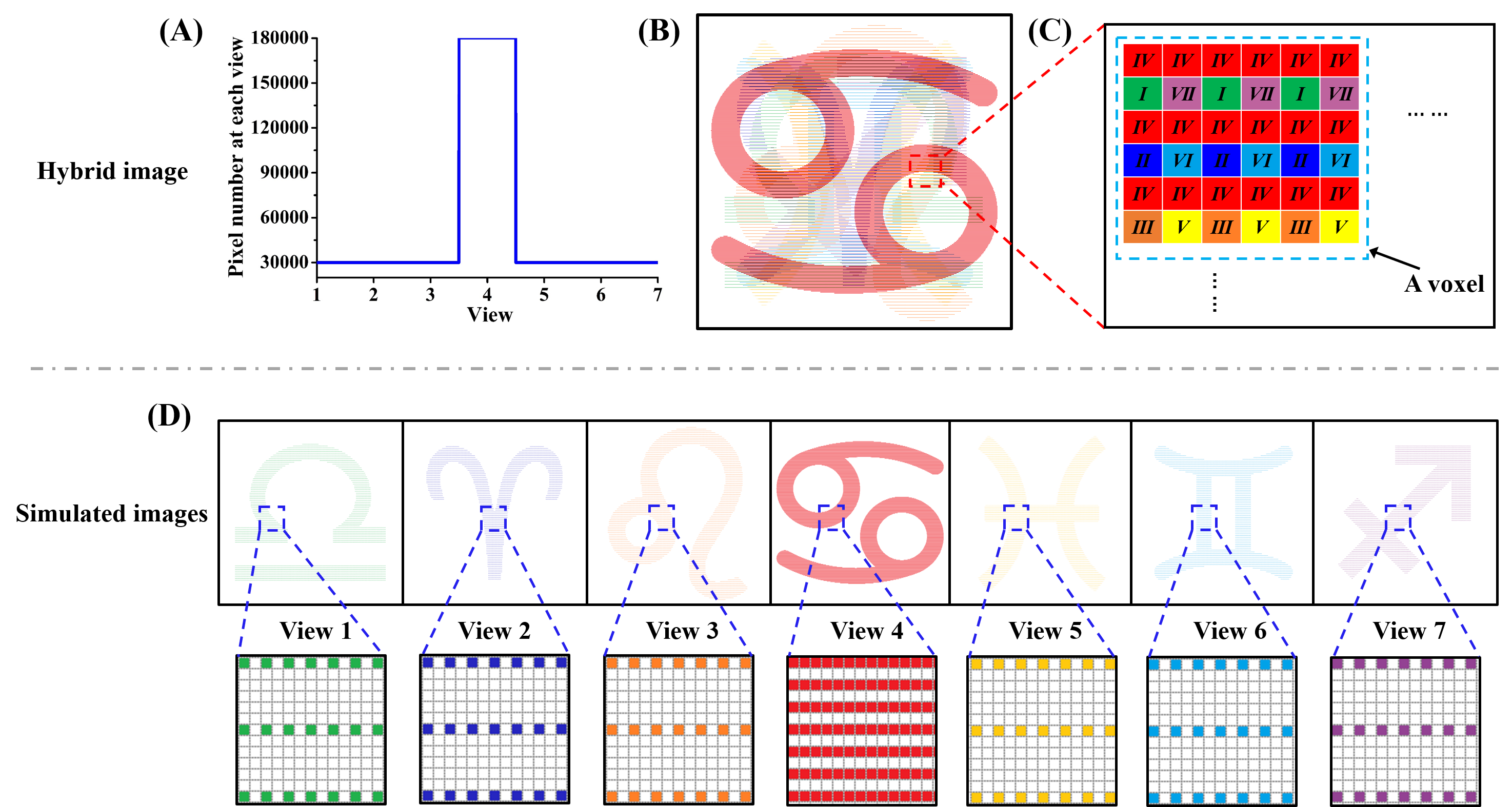


Figure S5. Schematic of the modulation method of spatial resolution both in the horizontal and vertical direction. (A) distribution of pixel number at each view. (B) the hybrid image which contains seven parallax images. (C) the arrangement of pixels in the voxels. There are 6 × 6 pixels in one voxel. (D) the simulated images observed at each view.

**Section S3: Experiment for obtaining 3D images from different perspectives**

**Figure S6** shows the photo of experimental devices that used to obtain 3D images from different views. Firstly, a white lamp is adopted to illuminate the 3D display prototype. Then a curved sliding rail is installed in front of the prototype. Finally a camera (D810, Nikon) is stabilized on the sliding rail. Pictures are taken from different perspectives by sliding the camera along the rail.

**
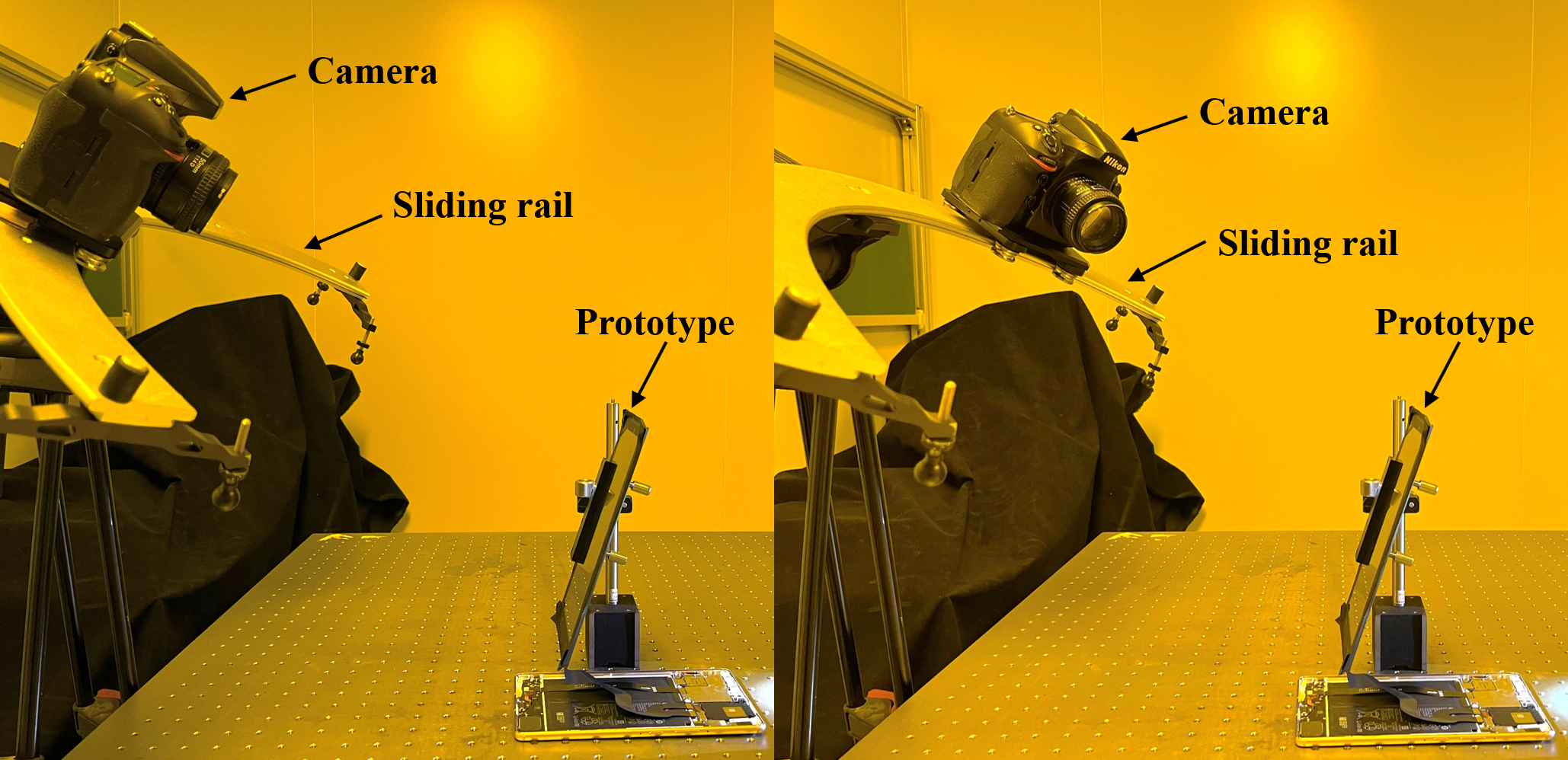
**

Figure S6. Experimental setup for obtaining 3D images from different persperctives.
